# Piperine improves the sensitivity of osteosarcoma cells to doxorubicin by inducing apoptosis and inhibiting the PI3K/AKT/GSK-3β pathway

**DOI:** 10.1186/s13018-023-03642-7

**Published:** 2023-03-09

**Authors:** Yubin Qi, Lin Yao, Jianke Liu, Wen Wang

**Affiliations:** 1 Department of Orthopedic Surgery, Qilu Hospital of Shandong University, No.107, Wenhua Xilu, Jinan, Shandong Province China; 2 Department of Orthopedic Surgery, The First Affiliated Hospital of Shandong First Medical University and Shandong Provincial Qianfoshan Hospital, No.16766, Jingshi Road, Jinan, Shandong Province China; 3 Department of Hand and Foot Surgery, The Jinxiang Hospital Affiliated to Jining Medical College, No.117, Jinfeng East Road, Jinxiang County, Shandong Province, China

**Keywords:** Piperine, Doxorubicin, Osteosarcoma, PI3K/AKT/GSK3β

## Abstract

**Background:**

Osteosarcoma is a primary bone malignancy associated with the highest incidence rate. Chemotherapy for osteosarcoma has not substantially changed, and survival of patients with metastatic tumours has reached a plateau. Doxorubicin (DOX) is a broad-spectrum anti-osteosarcoma drug; however, its application is limited due to its high cardiotoxicity. Piperine (PIP) has been verified to drive certain cancer cell death and increases chemosensitivity of DOX. However, the effects of PIP in promoting the chemosensitivity of osteosarcoma to DOX have not been studied.

**Methods:**

We examined the combined effect of PIP and DOX on U2OS and 143B osteosarcoma cells. CCK-8 assays, scratch assays, flow cytometry analysis, and western blotting were performed. Furthermore, the effect of PIP combined with DOX on osteosarcoma tumours was observed in vivo using nude mice.

**Results:**

PIP can increase the chemosensitivity of U2OS and 143B cells to DOX. Both in vitro and in vivo results showed the dramatic inhibition of cell proliferation and tumour growth by the combined therapy group compared to monotherapy groups. Apoptosis analysis revealed that PIP augments DOX-induced cell apoptosis by upregulating BAX and P53 expression, as well as reducing Bcl-2 expression. Furthermore, PIP also attenuated the initiation of the PI3K/AKT/GSK-3β signaling pathway in osteosarcoma cells by altering the expression levels of P-AKT, P-PI3K and P-GSK3β.

**Conclusions:**

This study revealed for the first time that PIP can potentiate the sensitivity and cytotoxicity of DOX during osteosarcoma therapy in vitro and in vivo*,* which probably achieved by inhibiting the PI3K/AKT/GSK-3β signalling pathway.

## Background

Osteosarcoma is one of the most common malignant bone tumours, accounting for approximately 55% of all bone malignancies. Its incidence rate in children and adolescents is second only to lymphoma and brain tumour. Within the 15–19 years age group in the general population, the incidence of osteosarcoma has increased from 2–3 million to 8–11 million per year [1]. Comprehensive therapy, including pre-surgical chemotherapy, surgical resection, and post-surgical chemotherapy, has increased the five-year survival rate from 20 to 70% of patients suffering from limited osteosarcoma [2]. However, acquired drug resistance associated with poor prognosis can be observed in osteosarcoma patients with other organ metastasis or recurrent, with a survival rate of 20% for five years, and the recurrence rate remains high at approximately 35% [3]. Chemotherapy for osteosarcoma has not substantially changed during the past 20 years, and patient survival in metastatic tumours has reached a plateau, with continued unsatisfactory results [4, 5]. Therefore, the efficacy of osteosarcoma treatment should be improved urgently. The search for efficacious therapy agents that increase chemotherapy response is important for improving the prognosis of osteosarcoma patients.

Doxorubicin (DOX), a naturally occurring anthracycline antibiotic, is a topoisomerase II inhibitor and a long-standing drug for chemotherapy. It can effectively treat solid tumours by causing a DNA damage response and blocking the G2/M-phase cell cycle, inducing apoptosis [6]. It remains the backbone of several cancer therapies, including osteosarcoma. Despite the considerable antitumour activity of DOX, its limited dose determines its therapeutic effectiveness owing to its intense cardiotoxicity [7]. Cardiotoxicity has been reported to limit successful cancer treatment, with cardiomyopathy occurring in approximately 50–60% of patients receiving high doses of DOX [8, 9]. To optimize therapy for maximum effectiveness while minimizing side effects, DOX is often used in combination with other drugs [10, 11]. New drugs are therefore urgently needed to improve the toxic effect and minimize the dosage and adverse reaction of DOX.

Piperine (PIP; 1-[5-[1,3-phenyldioxy-5-yl]-1-oxo-2,4,pentadienyl) is the most prevalent alkaloid in the diet and is particularly found in berries and roots of *Piper nigrum* and *Piper longum* [12]. Its immunomodulatory, hepatoprotective, anti-inflammatory, antibacterial, and ulcerative functions have been elucidated [13–15]. In vivo and in vitro studies on breast, prostate, colon cancers have shown that PIP can induce cell cycle arrest, increase autophagy coupled with apoptosis, and disrupt redox balance, which directly or indirectly affects the survival of tumour cells and inhibits invasion and distant metastatic processes with no significant detrimental impact on healthy cells [16–20]. More importantly, Zhang et al. [21] showed that PIP can obviously hinder the growth of HOS and U2OS osteosarcoma cells, while exhibiting lower cytotoxic activity against normal human hFOB osteoblasts.

The therapeutic potential of PIP for various cancers is under investigation, but its role with DOX treatment of osteosarcoma cells remains to be elucidated. Thus, we aimed to analyze the combined impact of PIP and DOX on the viability and migration capacity of osteosarcoma U2OS and 143B cells and understand the mechanism involved.

## Materials and methods

### PIP and DOX preparation

PIP (Puffe Biotechnology, Beijing, China) and DOX (BoMei Biotechnology, HeFei, China) were dissolved in DMSO and culture medium at an initial concentration of 2.85 mg/ml and 0.1 mg/ml, respectively, and stored at − 20 °C. Both were diluted in PBS as working concentrations.

### Cell culture

U2OS and 143B cells were acquired from Procell (Wuhan, China). Cells were separately incubated in complete McCoy^’^s 5A/MEM (ServiceBio, Wuhan, China) containing penicillin, streptomycin (ServiceBio, WuHan, China), and 10% foetal bovine serum (Biosharp Technology, Hefei, China), at 37 °C and 5% CO_2_.

### Cytotoxicity assay and combination index

Cell viability was assessed using the CCK-8 assay. Briefly, 1 × 10^5^ cells were inoculated in 96-well plates and cultivated for 24 h. Various concentrations of PIP (0, 14, 28, 56, 112, 224 µg/ml) or DOX (0, 0.25, 0.5, 1, 2, 4 µg/ml) were then added, and the cells were cultured for 24 or 48 h. In the combination therapy trial, cells were co-cultured in different concentrations of PIP and DOX as described above for 24 or 48 h. The CCK-8 assay kit (Apex Bio, Houston, TX, USA) was used to detect the influence of the pharmacological agents on cell survival. Briefly, each well was supplemented with 10 μL CCK-8, avoiding bubbles, and incubated at 37 °C for 3 h. The samples were mixed using a shaking platform before measuring the optical density (OD) at 450 nm of each well using a multifunction full wavelength scanner (BioRad, Hercules, CA, USA). Percent cell viability was calculated as follows:

$${\text{Cell}}\;{\text{viability }} = \frac{{{\text{OD}}_{{{\text{Test}}}} - {\text{OD}}_{0} }}{{{\text{OD}}_{{{\text{Con}}}} - {\text{OD}}_{0} }} \times 100\%$$

IC_50_ obtained through SPSS Statistics 21 (IBM Corp, NewYork, USA). Coefficient of drug interaction (CDI) is calculated using the following formula, CDI = AB/(A × B). The ratio of the absorbance of the combined drug group to that of the control group is defined as AB, A and B refers to the ratio of absorbance of the single drug group to the control group. CDI values < 1, = 1, > 1 represent synergistic, additive and antagonistic effects, respectively.

### Scratch assays

Cells (4 × 10^5^/well) were inoculated into a 6-well plate and incubated at 37 °C and 5% CO_2_ until the cells were approximately 90% confluent. The cell layer on the base of the plate was then scratched using a 100-μL micropipette tip. Subsequently, the suspended cells at the scratch were washed with PBS. Cells were further incubated in serum-free medium with various treatments (cell culture medium, PIP [56 µg/ml], DOX [1 µg/ml], and PIP + DOX [56 µg/ml and 1 µg/ml, respectively]) for 24 h. Photographed under the microscope at 12 and 24 h after the operation and the scratched area was measured using Image J software (1.53e National Institutes of Health, USA). Cell migration rate was calculated as below: Migration rate = (1 − scratch width after cell migration and culture/initial scratch width) × 100%

### Apoptosis assays

U2OS and 143B cells (1 × 10^5^/ml) were grown in 6-well plates for 24 h and incubated with cell culture medium, PIP (56 µg/ml), DOX (1 µg/ml), or PIP + DOX (56 µg/ml and 1 µg/ml, respectively). After 48 h of stimulation, cells were harvested via centrifugation, directly used in the Annexin V-FITC Apoptosis Assay Kit (Servicebio, WuHan, China), and assayed in a FACSAria Cell Sorter (Becton, BD Bio, San Jose, CA, USA). The apoptosis rate was measured and analyzed using the FlowJo software version V10.5.2 (BD Biosciences, Franklin Lakes, NJ, USA).

### Western blot assay

Cells were treated with cell culture medium, DOX(1 µg/ml), PIP (56 µg/ml), or PIP + DOX (56 µg/ml and 1 µg/ml, respectively) for 48 h. Adherent cells were digested with trypsin, washed with PBS and then 200ul RIPA was added and centrifuged at 12,000r for 15 min at 4 °C. The BCA protein assay kit (Beyotime Biotechnology) was used to determine the protein quantification of each group. Total protein (30 μg) was loaded onto a 10% gel, detached with SDS-PAGE and transmitted to a PVDF membrane (Millipore, Burlington, MA, USA) before blocking with blocking buffer (Beyotime Biotechnology) for 1.5 h at 20 °C. Primary antibodies(1:1000) against Bax (Affinity Biosciences Cat# AF0120), AKT (Affinity Cat# AF6261), Bcl-2 (Affinity Cat# AF6139), p-AKT (Ser473 Affinity Cat# AF0016), p-PI3K (Affinity Cat# AF3241), P53 (Affinity Cat# AF0879), and p-GSK3β(Affinity Cat# AF2016) were diluted with primary antibody dilution buffer(Beyotime Biotechnology).The membranes were washed thrice before incubating with the above non-conjugated primary antibodies at 4 °C overnight. A secondary antibody (1:5000) was added and incubated at 20 °C for 2 h, before placing on a decolorizing shaker and washing in TBST for three times. The ECL luminescent solution (Millipore; wbkls0100) was used for visualization, and Chemidoc MP gel imaging system (BioRad) was used to display the expression of target protein. Images were captured and data were analyzed using Image J software.

### In vivo animal experiment

Female Balb/c athymic mice (5–6 weeks old) were purchased from Vital River Laboratory Animal Technology (Beijing, China).This study was approved by the ethics committee of the First Affiliated Hospital of Shandong First Medical University (no. SYDWLS 2022014, 2022-05-10). All animal experimental practices were in accordance with the National Research Council's Guide for the Care and Use of Laboratory Animals. The right axilla (forearm) of each mouse was injected with 5 × 10^7^ 143B cells (log phase) suspended in 0.15 ml PBS. When tumors grew to approximately 100 mm^3^, rats were randomized into four groups (*n* = 5): control (saline), PIP (30 mg/kg), DOX (3 mg/kg), and PIP + DOX (30 mg/kg and 3 mg/kg, respectively). PIP was administered intraperitoneally once a day, while DOX was administered intraperitoneally every three days. The same volume of saline was injected intraperitoneally daily in the controlled group. Vernier callipers were used to conduct tumour size survey on third day basis. Tumour volumetric calculation was calculated as follows:

$${\text{Tumour}}\;{\text{volume }} = 0.5 \times L \times W^{2}$$

*L* and *W* were the long and short diameter of the tumour.The experiment was terminated when the tumour volume reached up to 2000 mm^3^. Tumour-bearing mice were weighed, and the primary tumour of each mouse was removed for further study.

### Statistical analyses

Differences between the two groups were compared using Student's t-test or one-way ANOVA. The Tukey multiple comparison test was used to compare more than two groups. All statistical analyses were performed using the GraphPad Prism 8.0 software. Data are shown as mean ± SD, and a group of representative data or images were selected for presentation. *P* < 0.05 indicated a significant difference.

## Results

### PIP improves the sensitivity of osteosarcoma cancer cells to DOX

**Fig. 1:**
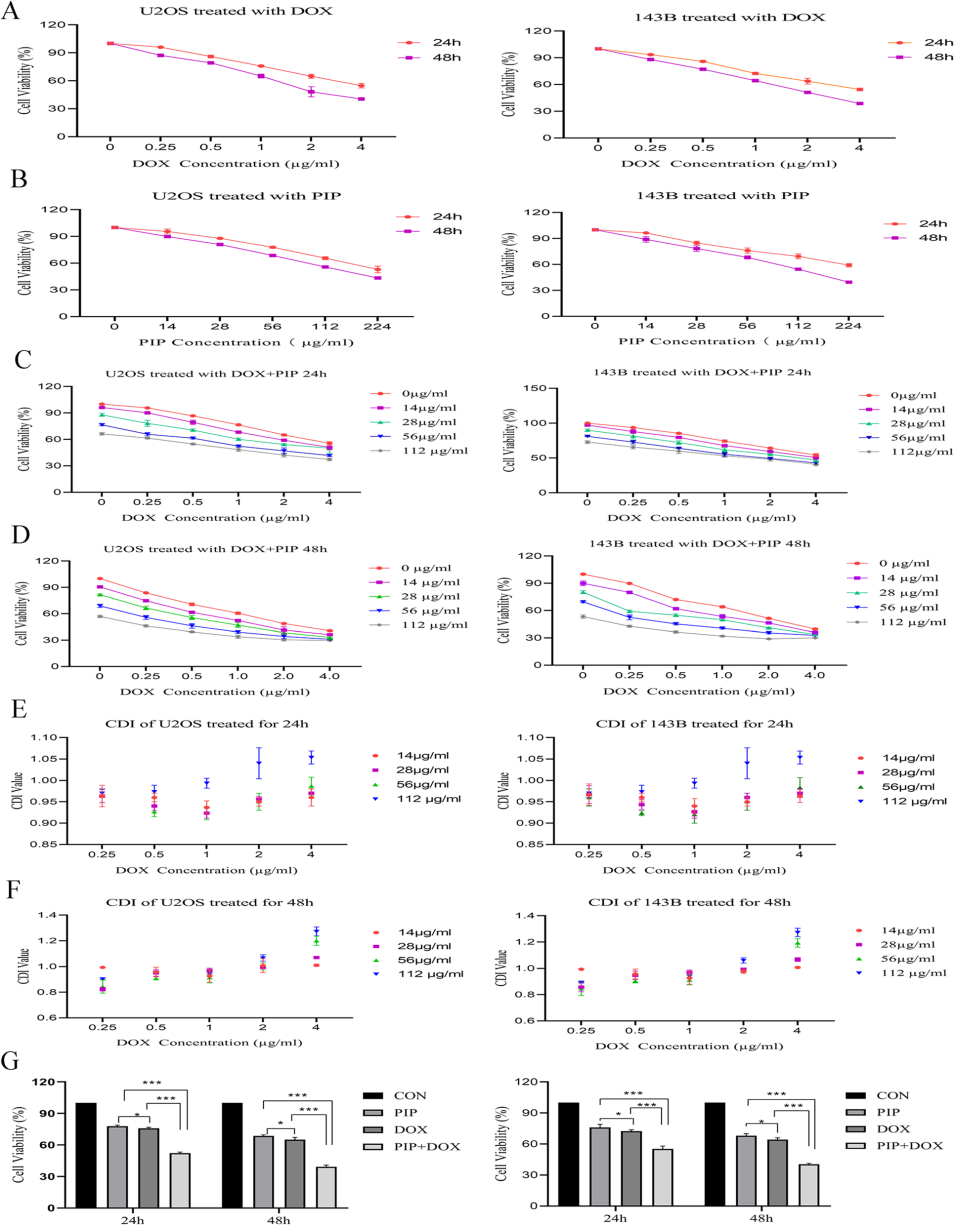
Effect of combination of PIP and DOX on proliferative activity of osteosarcoma cells. **A**, **B** Cytotoxicity analysis of PIP and DOX against U2OS and 143B cell. Varying concentrations of PIP combined DOX were observed to be potent in downregulating the cell viability of U2OS and 143B for 24 h and 48 h via the CCK8 assay. **C**, **D** Varying concentrations of PIP combined DOX could effectively decrease the cell viability of U2OS and 143B for 24 h and 48 h via the CCK8 assay. **E**, **F** CDI of different PIP combined with DOX for 24 h and 48 h in U2OS and 143B cells. **G** The PIP + DOX group was more efficient in inhibiting cell viability compared to the DOX group for 24 h and 48 h. *P < 0.05, **P < 0.01, ***P < 0.001

The CCK-8 assay revealed the dose- and time-dependent inhibitory impact of PIP therapy on osteosarcoma cell proliferation compared to the control (Fig. 1A, B). The IC_50_ values for U2OS cells treated with different concentrations of DOX for 24 h or 48 h were 5.5 µg/ml and 2.19 µg/ml, respectively, and the IC_50_ values for PIP were 302 µg/ml and 158.49 µg/ml; When 143B cells were treated with DOX at different concentrations for 24 h or 48 h, the IC_50_ values were 5.25 µg/ml and 2.17 µg/ml, respectively, and the IC_50_ values of PIP were 457.09 µg/ml and 138.04 µg/ml, respectively.The dose-dependent toxic effect of DOX was greater than that of PIP. We performed various concentrations of PIP in combination with DOX to culture U2OS and 143B cells and calculated the CDI value, found a synergistic effect of PIP and DOX at 24 h and 48 h, but the effect of their combination diminished above the IC_50_ concentration(Fig. 1C–F).The follow-up experimental study was carried out close to half the IC_50_ concentration-DOX(1 µg/ml)and PIP(56 µg/ml),and the synergistic effect of this combination was most pronounced and the combination therapy of PIP and DOX reduced cell proliferation by 97.96 ± 4.87% and 74.70 ± 7.45%, and 63.68 ± 2.95% and 64.19 ± 14.18% in U2OS and 143B cells for 24 h and 48 h, respectively, compared to the Dox monotherapy group(Fig. 1G). The results showed that the combined therapy of PIP and DOX was more efficacious than the single treatment of PIP and DOX; PIP improved the sensitivity of osteosarcoma cells to DOX.

### PIP enhances the inhibitory effect of DOX against osteosarcoma cell migration

**Fig. 2:**
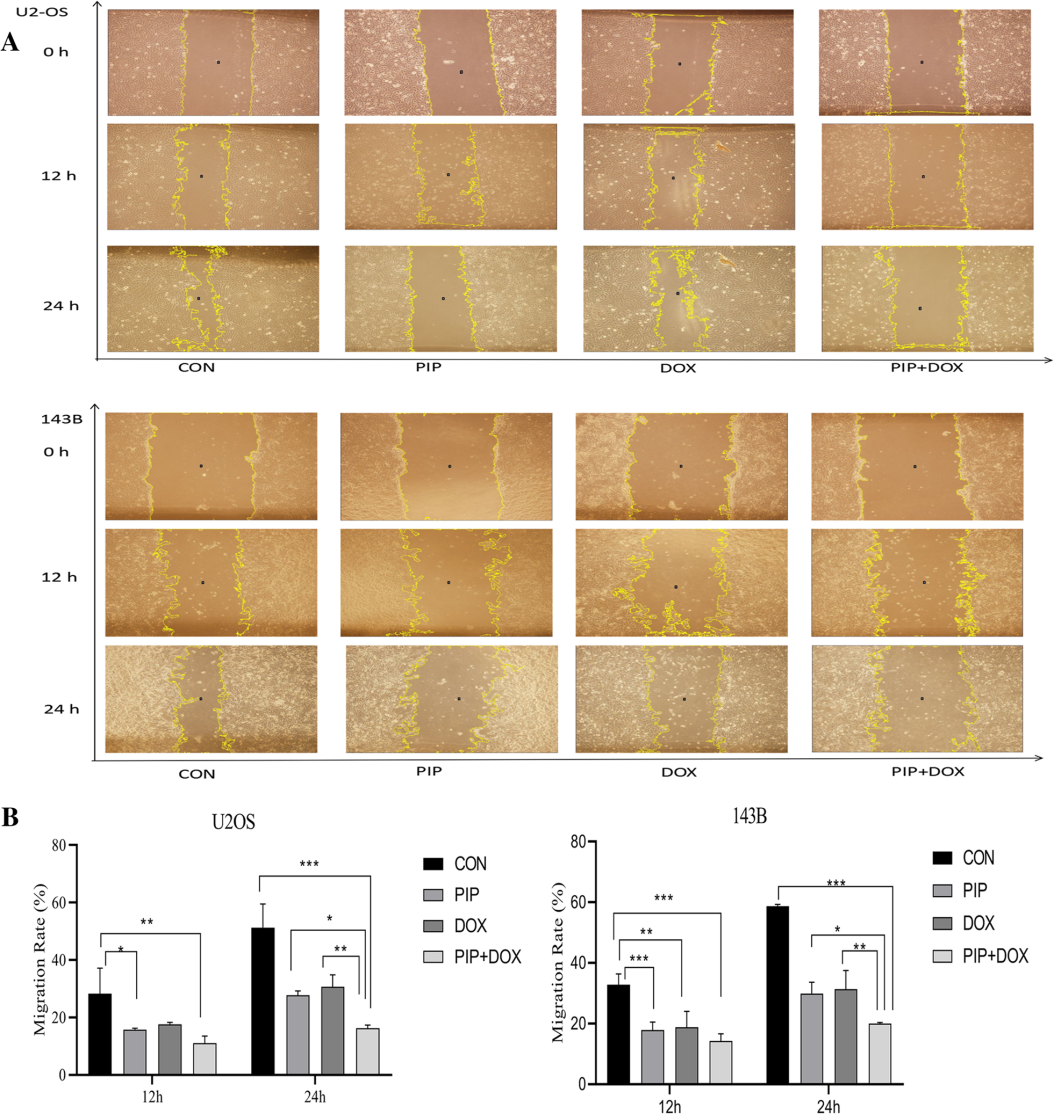
Effect of PIP combined with DOX on scratch healing in osteosarcoma cells. Cells were scratched using a 100 μL tip and then treated with PBS, PIP, DOX, and PIP + DOX. Scratch healing was observed at 0, 12 and 24 h after scratching. **A** Wound healing area of U2OS and 143B cells in each treatment group. **B** Quantification of migration rates. *P < 0.05, **P < 0.01, ***P < 0.001

Both PIP and DOX significantly inhibited U2OS and 143B cell migration compared to the control, and inhibition of cell migration was significantly enhanced when PIP was combined with DOX (Fig. 2). After incubation for 24 h, the proportion of the remaining wound area in cells treated with PIP + DOX group was dramatically higher compared to the other three groups, at 12 h; however, there was no significant difference between the three drug groups and all had more wound area than the control group. Thus, the combination therapy group may significantly decrease the number of growing and migrating cells.

### PIP enhances the sensitivity of osteosarcoma cells to DOX and induces an increase in apoptosis

**Fig. 3:**
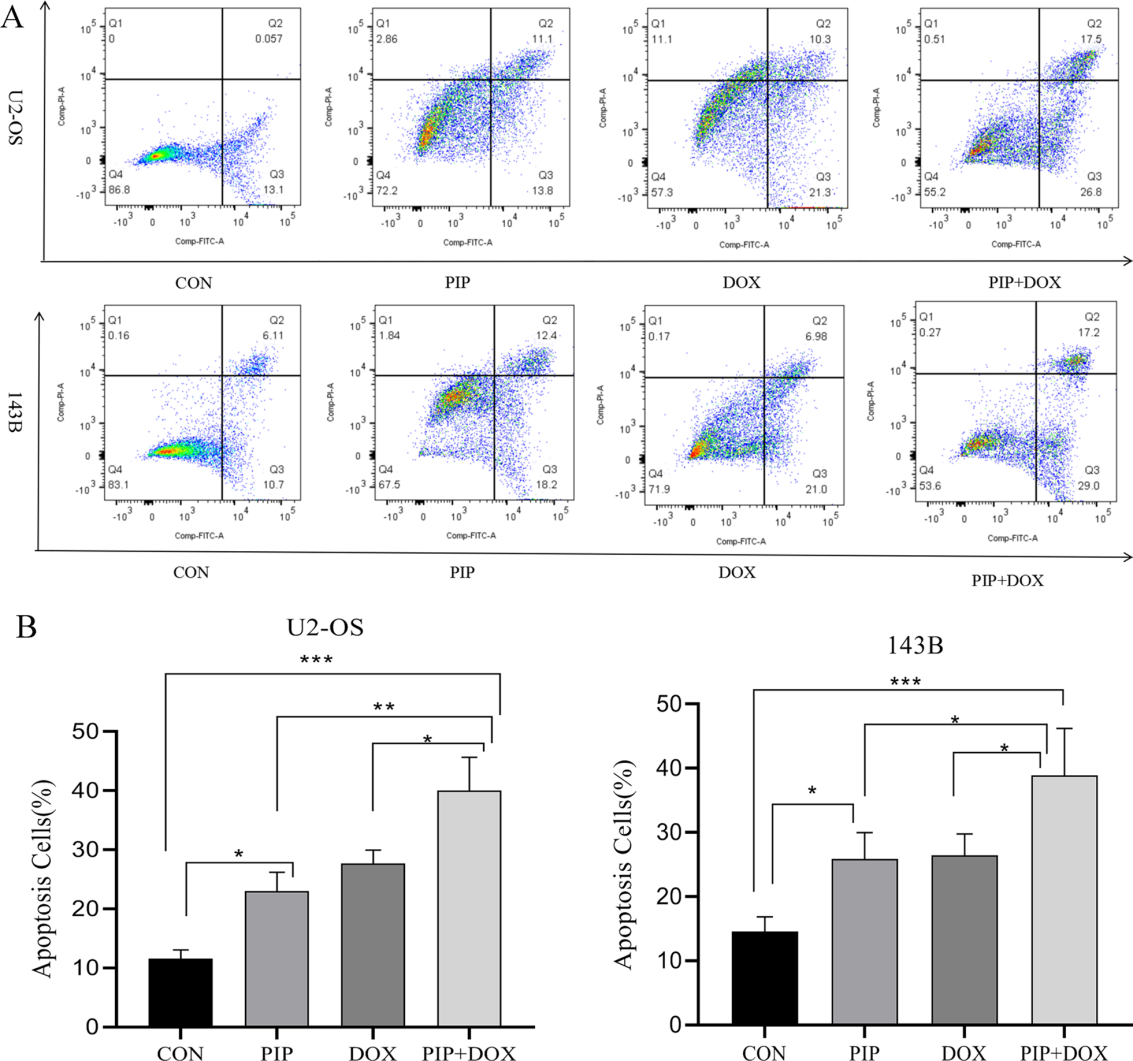
PIP combined with DOX promote apoptosis in osteosarcoma cells. **A** U2OS and 143B cells were treated with PIP, DOX, and PIP + DOX for 48 h. Apoptosis was determined using the Annexin V-FITC Flow Cytometry. **B** Quantification of apoptotic cells. *P < 0.05, **P < 0.01, ***P < 0.001

Compared with the control group, the apoptosis of U2OS and 143B cells was significantly induced (*P* < 0.05), when treated with PIP, DOX, or PIP + DOX for 48 h. Induction of apoptosis in osteosarcoma U2OS and 143B cells by the combination of PIP and DOX was superior to that in the control-, PIP-, or DOX-treated cells, their apoptosis rates increased by 44.4% and 47.2%, respectively, compared to DOX alone,and the apoptosis of 143B cells was more pronounced when treated with the combination of drugs (Fig. 3).

**Fig. 4:**
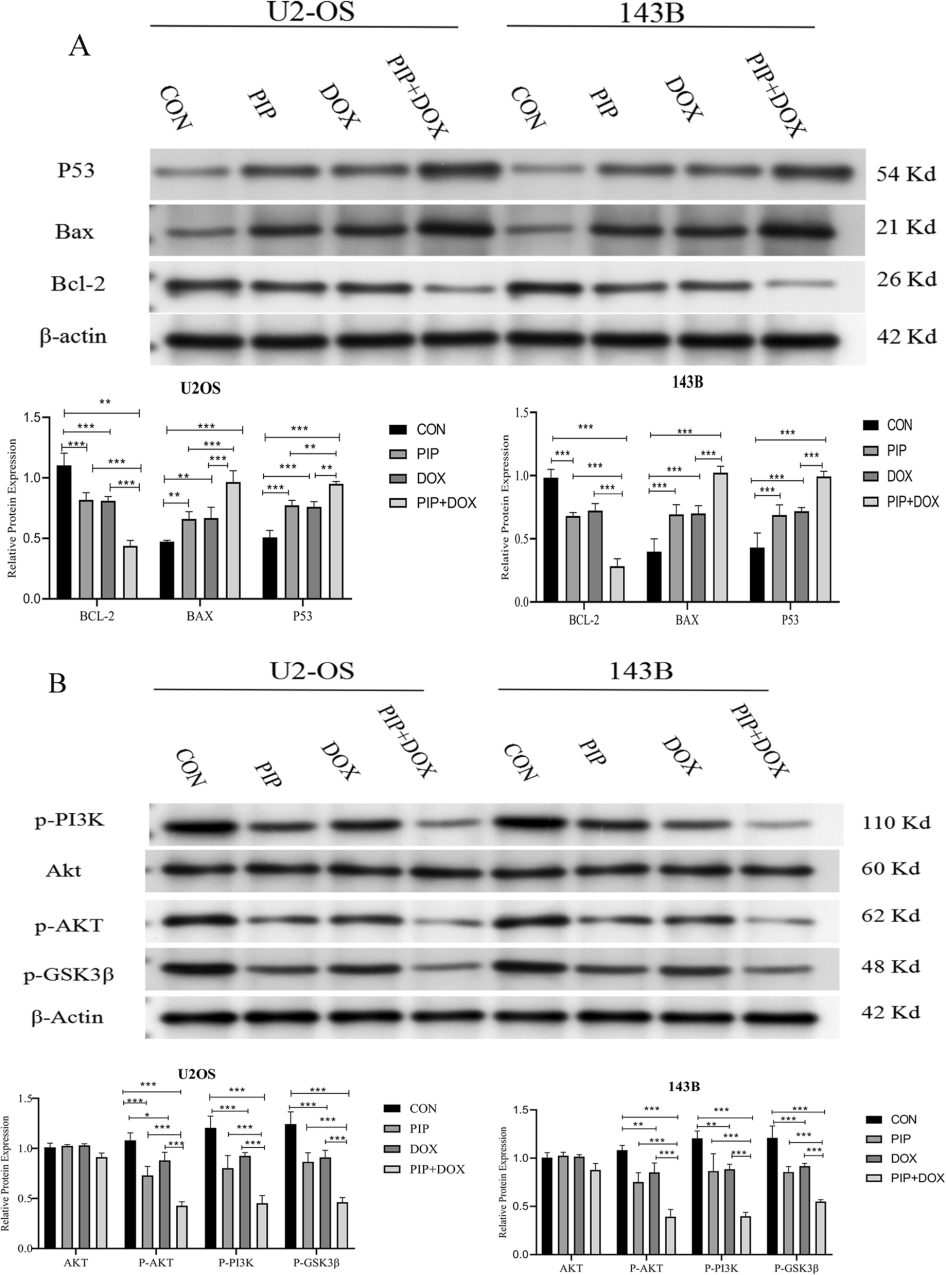
Effect of PIP combined with DOX on the PI3K/AKT/GSK3β signalling pathway. U2OS and 143B cells were treated with PIP, DOX, and PIP + DOX for 48 h. The protein was extracted, and the target protein expression was observed using western blot. **A** Relative expression of Bax, Bcl-2, and P53 in different groups. **B** Relative expression of AKT, p-AKT, p-PI3K, p-GSK3β. *P < 0.05, **P < 0.01

Western blotting suggested that the level of Bax and P53 expression was dramatically higher when PIP was combined with DOX than when either drug was used alone. In addition, we discovered that U2OS and 143B osteosarcoma cells exposed to PIP combined with DOX exhibited a remarkable lower expression of apoptosis-resistant proteins Bcl-2 (Fig. 4A). Collectively, these findings suggest that the PIP significantly promotes the apoptotic effect of DOX on osteosarcoma cells maybe by sensitizing P53/Bcl-2/Bax pathway.

### PIP increases the sensitivity of osteosarcoma cells to DOX by weakening the PI3K/AKT/GSK3β pathway

Inhibition of the PI3K/AKT signaling pathway is effective in reducing the proliferation of osteosarcoma cells, and therefore attenuation of this signaling pathway has been suggested as a target for tumour therapy. In our study, we demonstrated that PIP pretreatment enhanced the proliferation inhibition of osteosarcoma by DOX, and in order to clarify the mechanism of action, we performed Western Blot to detect the expression of proteins related to PI3K/AKT signaling pathway. The expression levels of p-AKT, p-PI3K, and p-GSK3β were considerably lowered in the PIP or DOX treatment group against the control group, but were more markedly reduced in the PIP + DOX group, whereas no considerable difference in AKT presentation levels was seen in all groups (Fig. 4B). In conclusion, our results suggested that PIP improved the chemosensitivity of osteosarcoma cells to DOX by weakening the PI3K/AKT/GSK3β signaling pathway.

### PIP potentiates the anti-tumour effect of DOX in an in situ osteosarcoma mouse model

**Fig. 5:**
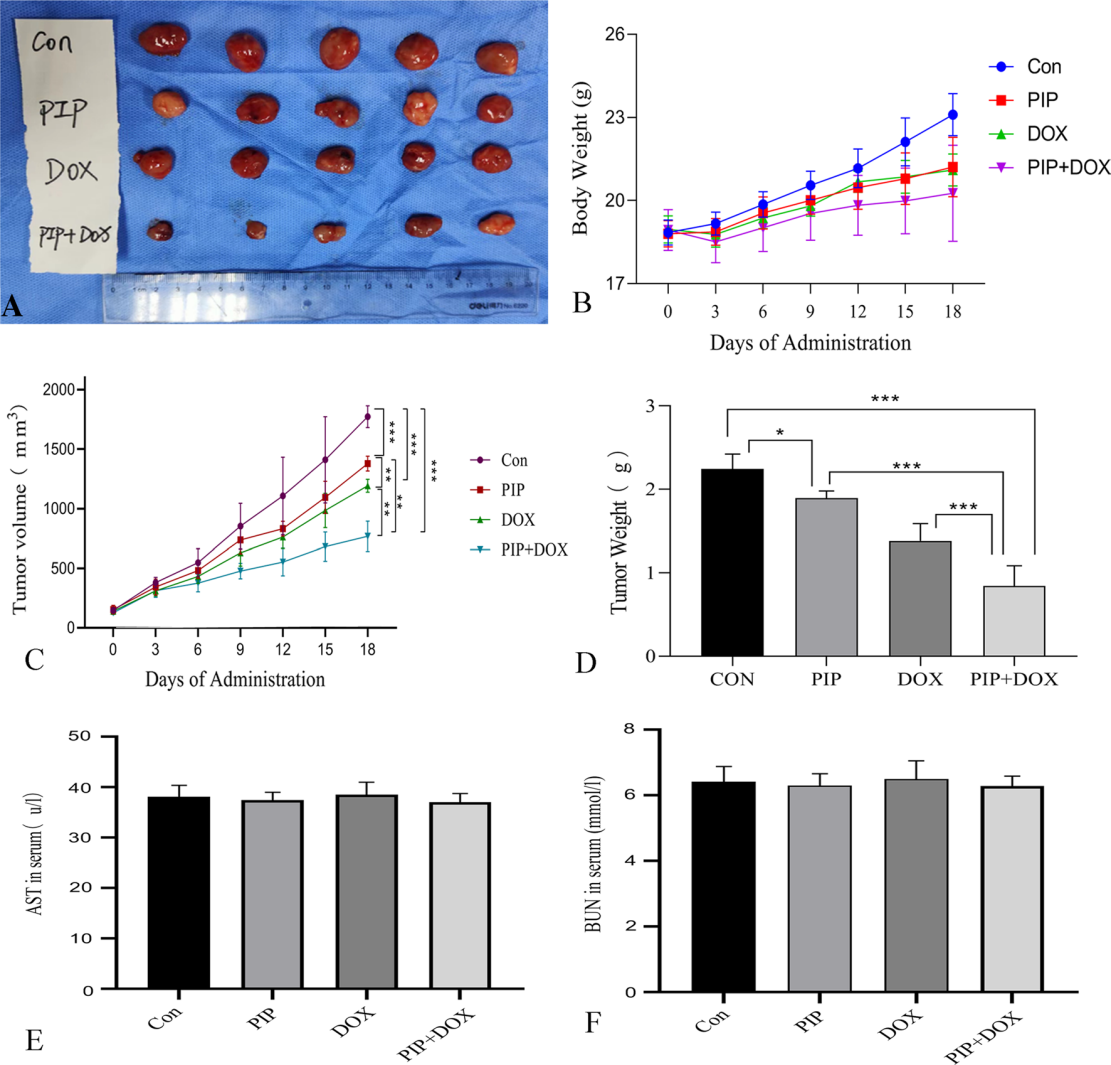
Anti-osteosarcoma effects of PIP, DOX, and PIP + DOX in vivo using mice models. Balb/c mice were randomly grouped and injected intraperitoneally with saline, PIP (30 mg/kg), DOX (3 mg/kg), and PIP + DOX (30 mg/ kg and 3 mg/kg, respectively) for 18 days. **A** Images of various groups of tumours. **B** Variations in body weight of mice in different groups per three days after administration. **C** Tumour volume measured every three days. **D** Tumour weight in each group after isolation. **E** AST and BUN in serum for each group. *P < 0.05, **P < 0.01, ***P < 0.001

After 18 consecutive days of drug injection intraperitoneally, tumour growth was remarkably inhibited in PIP- and DOX-treated mice compared to the saline-treated mice. Notably, a significant increase in the anti-tumor effect of the PIP + DOX treatment group was observed compared to the DOX or PIP treatment group (Fig. 5 A, C). We had observed no signs of lethargy, anorexia, or diarrhoea in mice treated with the combination of PIP and DOX. Dynamic observation of the mouse body weight revealed different trends in body weight change across the four groups. The saline-treated mice showed a linear increase, PIP- and DOX-treated mice showed a slow increase, and the PIP + DOX-treated group initially showed a modest decrease in body weight and a slow increase with further doses (Fig. 5B). The outcomes indicated that the tumour volume decreased by 22.26%, 37.27%, and 56.72%; and the tumour weight decreased by 15.06%, 38.59%, and 62.48% after 18 consecutive doses of PIP, DOX, and PIP + DOX, respectively, compared to the saline group (Fig. 5D). At the end of the experiment, we measured AST and BUN to assess hepatic and renal toxicity and found no significant differences between the groups, and the combination of PIP and DOX did not cause hepatic or renal damage (Fig. 5E, F).These results confirmed that PIP enhanced the anti-tumour effects of DOX.

## Discussion

We conducted in vivo and in vitro experiments on the combination of PIP and DOX. The results showed that both PIP and DOX inhibited the proliferation of osteosarcoma U2OS and 143B cells in vitro and promoted apoptosis, while the combination of PIP and DOX was significantly more effective than the single drug group in both inhibiting cell proliferation and surviving apoptosis, which was also confirmed by in vivo experiments.

Osteosarcoma is a common type of cancer in orthopaedic clinics, and despite ongoing extensive research, no significant therapeutic improvements have been achieved in the last few decades [4]. Earlier findings have suggested that osteosarcoma is a highly variable cancer, both in its clinical presentation and on a cellular and molecular level [22]. Paradoxically, only four drugs, cisplatin, DOX, isocyclophosphamide, and methotrexate, are currently used to treat this cancer, and chemotherapy-induced toxicity remains an unresolved problem in osteosarcoma and other cancers [23, 24]. DOX, one of the gold-standard chemotherapeutic agents for osteosarcoma, is widely used in clinical applications. However, DOX is largely limited in clinical use owing to its severe dose-dependent cardiotoxicity, which can result in cardio-vascular disease and eventually progress to congestive heart failure [25, 26]. Therefore, it is essential to explore a therapeutic approach that can diminish DOX toxicity and boost its antitumour effect. PIP has been shown to reduce the cardiotoxic side effects of DOX [27]. Previous studies and our current research suggest that PIP inhibited the proliferation of osteosarcoma cells with a positive correlation between dose and time, and the anti-cellular proliferation potential of this agent has also been demonstrated in breast and gastric cancers [21, 28–30]. These results suggest that PIP may be a candidate drug molecule with tumour growth inhibitory effects. In the present study, we demonstrated that PIP promoted the therapeutic effect of DOX in U2OS and 143B osteosarcoma cells. Notably, PIP alone or combined with DOX dramatically reduced cell viability and migration capacity compared to DOX treatment, and both showed synergistic anticancer effects in U2OS and 143B cells, corroborating previous reports that PIP may sensitize a variety of cancer cells to DOX [31, 32].

Current strategies to eradicate cancer cells include promoting apoptosis by blocking the cell cycle or restraining cancer cell proliferation. Apoptosis is a critical defensive mechanism for eliminating cancer cells and halting their progression. The main mechanism for many antitumour agents is to promote apoptotic pathways through various apoptosis-related signals. Two main pathways are involved in the stimulation of apoptosis in tumour cells: endogenous mitochondrial pathway and exogenous membrane death acceptor pathway [33]. Regulation of the endogenous pathway is mediated by Bcl-2 family proteins, which essentially comprises the anti-apoptotic factor Bcl-2 and apoptosis-promoting factor Bax. Bcl-2 overexpression and Bax underexpression can lower the sensitivity of cancers to chemotherapeutic drugs and avoid apoptosis [34]. Furthermore, as a transcription factor, p53 can regulate cell cycle interruption, DNA repair, and apoptosis [35]. In this study, we observed that p53 and BAX exposure was greatly enhanced in the PIP + DOX treatment group, while Bcl-2 was decreased, compared to the DOX treatment group. Thus, PIP inhibited osteosarcoma development by downregulating Bcl-2 expression and upregulating P53 and Bax expression.

Activation of the PI3K/AKT/GSK3β signalling pathway has been shown to play an important role in oncogenesis and progression of osteosarcoma; and its modulatory pathways are closely related to cell proliferation, migration, cell cycle progression, genetic variation, autophagy, multidrug resistance, and apoptosis [36, 37]. In osteosarcoma cells, the PI3K/AKT pathway is activated. AKT, a key downstream factor of PI3K, phosphorylates a large variety of substrates. Activated p-AKT promotes the phosphorylation of the downstream protein BAX, dissociating BAX and Bcl-2, thereby upregulating Bcl-2 [38]. In this study, PIP significantly reduced the Bcl-2 protein expression level in both osteosarcoma cell lines. GSK3β is a soluble serine/threonine protein kinase in eukaryotic cells, which is downstream of PI3K/AKT pathway. Revitalized AKT phosphorylates serine at the N-terminal position of the GSK3β protein, which promotes dephosphorylation of downstream β-linked proteins and entry of dephosphorylated β-linked proteins into the nucleus to promote cell proliferation. In a previous study, restrained PI3K/AKT/GSK3β pathway was found to inhibit lung NSCLC cell invasion and migration [39]. Evidence also suggests that celastrol attenuates the growth of human colon cancer cells via blocking the PI3K/AKT/GSK3β pathway [40]. In the present study, combined PIP and DOX treatment lowered the expression of p-PI3K, p-AKT, and p-GSK3β proteins, suggesting that the combination suppressed the activation of PI3K, leading to a decrease in phosphorylated AKT and the inhibition of phosphorylation of GSK3β protein, thus inhibiting cell proliferation.

The combined effect of PIP and DOX was confirmed in vivo using nude mice. Our results indicated that combination of the two drugs for osteosarcoma has a stronger anti-cancer effect than the single-drug treatment, which corresponded to the results in vitro. Furthermore, the combination of PIP and DOX enhanced antitumour activity without increasing toxicity. And the study has some limitations, for example, protein expression can be explored using activators of the PI3K/AKT signalling pathway; the lung metastases can be further observed in vivo experiments and further clinical studies are needed to verify this hypothesis.

## Conclusion

In conclusion, this study is the first to reveal that the combination of PIP and DOX can potentiate the sensitivity and cytotoxicity of DOX in the therapy of osteosarcoma in vitro and in vivo. Furthermore, this combined treatment was more effective than treatment with PIP or DOX alone and can reduce the dosage of DOX by enhancing its antitumour effect. Additionally, this study has elucidated the molecular mechanism by which PIP improves the antitumour effects of DOX and provides a partial theoretical foundation for the comprehensive treatment of osteosarcoma in clinical settings.

## Data Availability

All the data and materials are available from the corresponding author via an e-mail.

## Abbreviations

TBST: Tris buffered saline with Tween-20
DMSO: Dimethyl sulfoxide
PBS: Phosphate buffered saline
PVDF: Polyvinylidene difluoride
CCK8: Cell counting kit 8
OD: Optical density
CDI: Coefficient of drug interaction
IC_50_: Half maximal inhibitory concentration
SDS-PAGE: Sodium dodecyl sulfate-polyacrylamide gel electrophoresis
AST: Aspartate transaminase
BUN: Blood urea nitrogen
